# 3-{[(Benz­yloxy)carbon­yl]amino}­butanoic acid

**DOI:** 10.1107/S1600536811035276

**Published:** 2011-09-03

**Authors:** Taira Kimino, Isao Fujii

**Affiliations:** a Department of Biological Science and Technology, Tokai University, 317 Nishino, Numazu, Shizuoka 410-0321, Japan; b School of Science, Tokai University, 4-1-1 Kitakaname, Hiratuka, Kanagawa 259-1292, Japan

## Abstract

In the title compound, C_12_H_15_NO_4_, the butyric acid group has a stretched *trans* conformation. The dihedral angle between the phenyl ring and the oxycarb­oxy­amino N—(C=O)—O—C plane is 56.6 (2)°. In the crystal, an inversion dimer is formed by a pair of O—H⋯O hydrogen bonds. The dimers are further linked by N—H⋯O hydrogen bonds between amide groups, forming a tape along the *b* axis.

## Related literature

For general background to 3-amino­butanoic acid, see: Cohen *et al.* (2011 ▶). For bond-length data, see: Allen *et al.* (1987 ▶). For structures of related metallo-organic compounds, see: Bryan *et al.* (1961 ▶); Böhm & Seebach (2000 ▶); Gross & Vahrenkamo (2005 ▶).
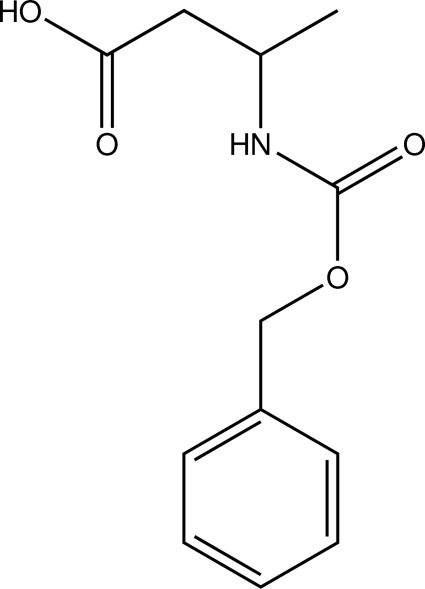


## Experimental

### 

#### Crystal data

C_12_H_15_NO_4_
*M* *_r_* = 237.25
Monoclinic, 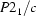

*a* = 23.1413 (7) Å
*b* = 4.9589 (4) Å
*c* = 11.0879 (6) Å
β = 103.075 (6)°
*V* = 1239.41 (13) Å^3^
*Z* = 4
Cu *K*α radiation
μ = 0.8 mm^−1^
*T* = 297 K
0.4 × 0.2 × 0.2 mm

#### Data collection

Enraf–Nonius CAD-4 diffractometer
Absorption correction: ψ scan (North *et al.*, 1968 ▶) *T*_min_ = 0.74, *T*_max_ = 0.856
2696 measured reflections
2547 independent reflections
1669 reflections with > 2σ(i)
*R*_int_ = 0.023
3 standard reflections every 300 reflections  intensity decay: none

#### Refinement

*R*[*F* ^2^ > 2σ(*F* ^2^)] = 0.046
*wR*(*F* ^2^) = 0.131
*S* = 1.02
2547 reflections
164 parameters
H atoms treated by a mixture of independent and constrained refinement
Δρ_max_ = 0.14 e Å^−3^
Δρ_min_ = −0.14 e Å^−3^

### 

Data collection: *CAD-4* Software (Enraf–Nonius, 1989 ▶); cell refinement: *CAD-4* Software; data reduction: *XCAD4* (Harms & Wocadlo, 1995 ▶); program(s) used to solve structure: *SHELXS97* (Sheldrick, 2008 ▶); program(s) used to refine structure: *SHELXL97* (Sheldrick, 2008 ▶); molecular graphics: *ORTEP-3* (Farrugia, 1997 ▶); software used to prepare material for publication: *PLATON* (Spek, 2009 ▶) and *WinGX* (Farrugia, 1999 ▶).

## Supplementary Material

Crystal structure: contains datablock(s) I, global. DOI: 10.1107/S1600536811035276/is2769sup1.cif

Structure factors: contains datablock(s) I. DOI: 10.1107/S1600536811035276/is2769Isup2.hkl

Supplementary material file. DOI: 10.1107/S1600536811035276/is2769Isup3.cml

Additional supplementary materials:  crystallographic information; 3D view; checkCIF report

## Figures and Tables

**Table 1:** Hydrogen-bond geometry (Å, °)

*D*—H⋯*A*	*D*—H	H⋯*A*	*D*⋯*A*	*D*—H⋯*A*
O1—H1⋯O2^i^	1.18 (3)	1.48 (3)	2.650 (2)	177 (2)
N1—H*N*1⋯O3^ii^	0.85 (2)	2.04 (2)	2.865 (2)	165 (2)

Symmetry codes: (i) 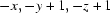; (ii) 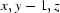.

**Fig. 1.**
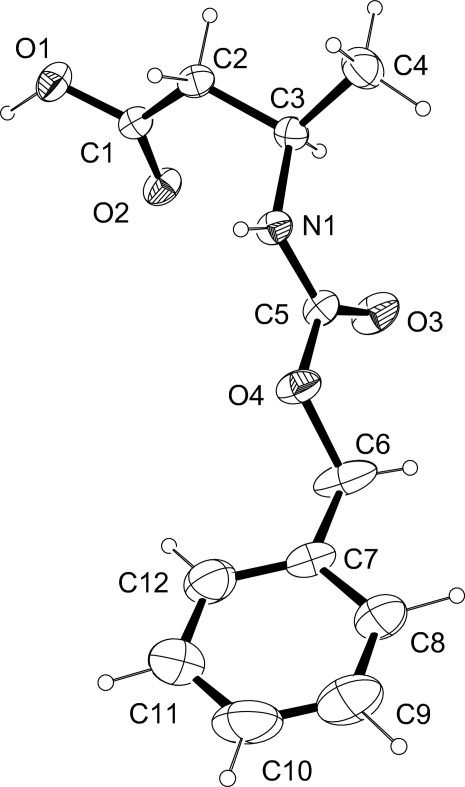
The molecular structure of the title compound. Displacement ellipsoids are drawn at the 30% probability level.

**Fig. 2.**
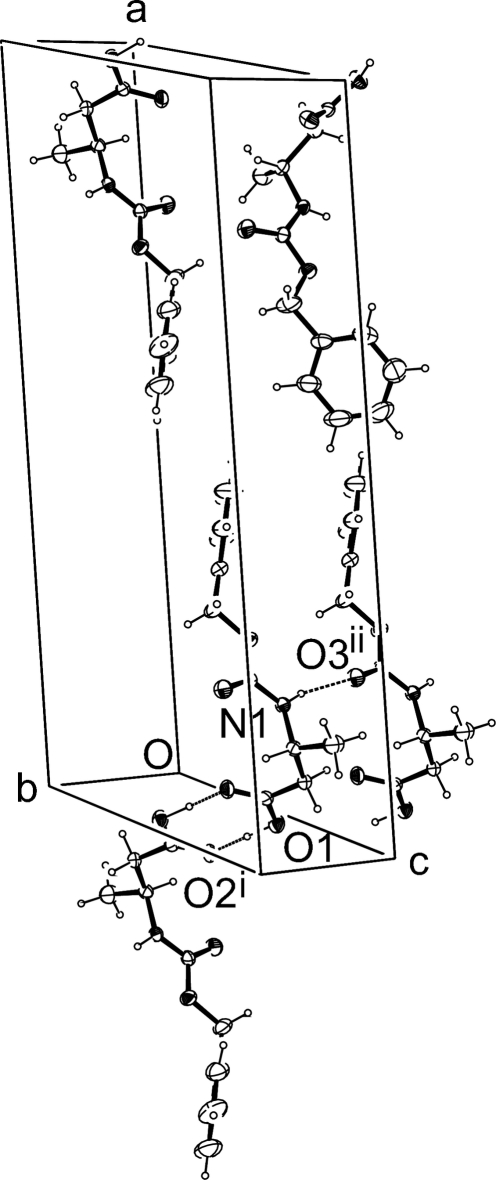
A packing view of the title compound. Dashed lines indicate O—H···O and N—H···O interactions [symmetry codes: (i) - x, 1 - y, 1 - z; (ii) x, y - 1, z].

## supplementary crystallographic information

### Comment

Solid-phase synthesis is now the accepted method for peptide synthesis, in which the protected natural or non-natural amino acids are widely used.  3-Aminobutanoic acid (BABA) is one of the non-protein amino acids, and it attracts attentions to building block.  At the same time, BABA potentially possesses various bioactivities.  Downy mildew of lettuce (*Bremia lactucae*) is a serious disease, but BABA is considered with one of the disease resistance inducers (Cohen *et al.*, 2011). Despite the agrichemical or pharmaceutical desires, crystal structures of BABA derivatives have not been cleared except for the structures of some metallo-organic compounds (Bryan *et al.*, 1961; Gross & Vahrenkamo, 2005; Böhm & Seebach, 2000) because of its difficulty in crystallization.

Fortunately, the title compound, 3-benzyloxycarbonylaminobutanoic acid (Cbz-BABA), (I), was crystallized, and we herein report on the crystal structure.  The molecular structure of (I) is shown in Fig. 1. The bond lengths (Allen *et al.*, 1987) and angles are within normal ranges except for the O-H bond length at the carboxy dimer. The part of BABA is essentially similar with that reported by Gross & Vahrenkamo (2005). The butyric acid group owns a stretched *trans*-conformation (O1-C1-C2-C3-C4). At the *β*-position the benzyloxycarboxyamino group is attached perpendicular to the butyric acid group.  The phenyl group is twisted against the least-squares plane of the oxycarboxyamino group (N1/C5/O3/O4/C6/C7) with the dihedral angle of 56.6 (2)°.

In the crystal structure, an enantiomer makes a planar structure with the intermolecular hydrogen bond (N1—HN1···O3) along the *b* axis. The planar structure is stacked to the enantiopure layer along the *c* axis.  The carboxy dimer is made from the enantiomeric isomers with the intermolecular hydrogen bond (O1—H1···O2). The H atom is shared by carboxy dimer then the bond distance O1—H1 is longer than that of general carboxy group. The hydrophobic and hydrophilic layers are well separated along the *a* axis.  The structure shows a herring bone stacking mode (Fig. 2).

### Experimental

The title compound was purchased from Aldrich-Sigma Co. Ltd. Rod-like colourless crystals suitable for X-ray diffraction were obtained by vapour-phase diffusion of an ethanol and chloroform mixture solution at 297 K.

### Refinement

All H atoms were located in a difference-Fourier map. H atoms bonded to N and O atoms were then refined isotropically. Other H atoms were positioned geometrically and refined using a riding model, with C—H = 0.95–0.99 Å and with *U*_iso_(H) = 1.2 (1.5 for methyl groups) times *U*_eq_(C).

### Crystal data

**Table:**

C_12_H_15_NO_4_	*F*(000) = 504
*M**_r_* = 237.25	*D*_x_ = 1.271 Mg m^−^^3^
Monoclinic, *P*2_1_/*c*	Cu *K*α radiation, λ = 1.54184 Å
Hall symbol: -P 2ybc	Cell parameters from 25 reflections
*a* = 23.1413 (7) Å	θ = 30.0–35.0°
*b* = 4.9589 (4) Å	µ = 0.8 mm^−^^1^
*c* = 11.0879 (6) Å	*T* = 297 K
β = 103.075 (6)°	Rod, colourless
*V* = 1239.41 (13) Å^3^	0.4 × 0.2 × 0.2 mm
*Z* = 4	

### Data collection

**Table:**

Enraf–Nonius CAD-4 diffractometer	*R*_int_ = 0.023
Radiation source: sealed X-ray tube	θ_max_ = 74.9°, θ_min_ = 2.0°
ω/2θ scans	*h* = −28→28
Absorption correction: ψ scan (North *et al.*, 1968)	*k* = −6→0
*T*_min_ = 0.74, *T*_max_ = 0.856	*l* = −13→0
2696 measured reflections	3 standard reflections every 300 reflections
2547 independent reflections	intensity decay: none
1669 reflections with > 2σ(i)	

### Refinement

**Table:**

Refinement on *F*^2^	Secondary atom site location: difference Fourier map
Least-squares matrix: full	Hydrogen site location: inferred from neighbouring sites
*R*[*F*^2^ > 2σ(*F*^2^)] = 0.046	H atoms treated by a mixture of independent and constrained refinement
*wR*(*F*^2^) = 0.131	*w* = 1/[σ^2^(*F*_o_^2^) + (0.0542*P*)^2^ + 0.3091*P*] where *P* = (*F*_o_^2^ + 2*F*_c_^2^)/3
*S* = 1.02	(Δ/σ)_max_ < 0.001
2547 reflections	Δρ_max_ = 0.14 e Å^−^^3^
164 parameters	Δρ_min_ = −0.14 e Å^−^^3^
0 restraints	Extinction correction: *SHELXL97* (Sheldrick, 2008), FC^*^=KFC[1+0.001XFC^2^Λ^3^/SIN(2Θ)]^-1/4^
Primary atom site location: structure-invariant direct methods	Extinction coefficient: 0.0020 (4)

### Special details

**Table:**

Geometry. Bond distances, angles etc. have been calculated using the rounded fractional coordinates. All su's are estimated from the variances of the (full) variance-covariance matrix. The cell esds are taken into account in the estimation of distances, angles and torsion angles
Refinement. Refinement on *F*^2^ for ALL reflections except those flagged by the user for potential systematic errors. Weighted *R*-factors *wR* and all goodnesses of fit *S* are based on *F*^2^, conventional *R*-factors *R* are based on *F*, with *F* set to zero for negative *F*^2^. The observed criterion of *F*^2^ > σ(*F*^2^) is used only for calculating -*R*-factor-obs *etc*. and is not relevant to the choice of reflections for refinement. *R*-factors based on *F*^2^ are statistically about twice as large as those based on *F*, and *R*-factors based on ALL data will be even larger.

### Fractional atomic coordinates and isotropic or equivalent isotropic displacement parameters (Å^2^)

**Table:**

	*x*	*y*	*z*	*U*_iso_*/*U*_eq_	
O1	0.00381 (6)	0.2239 (3)	0.60261 (14)	0.0693 (5)	
O2	0.05705 (6)	0.5990 (3)	0.61469 (14)	0.0671 (5)	
O3	0.20820 (7)	0.8467 (3)	0.78378 (18)	0.0841 (7)	
O4	0.25967 (6)	0.5196 (3)	0.71327 (15)	0.0686 (5)	
N1	0.18104 (7)	0.4105 (3)	0.78288 (15)	0.0513 (5)	
C1	0.04634 (8)	0.3818 (4)	0.65874 (18)	0.0501 (6)	
C2	0.07918 (8)	0.2819 (4)	0.78120 (17)	0.0536 (6)	
C3	0.13228 (8)	0.4484 (4)	0.84471 (16)	0.0511 (6)	
C4	0.15096 (11)	0.3763 (6)	0.9808 (2)	0.0862 (9)	
C5	0.21503 (8)	0.6116 (4)	0.76220 (18)	0.0536 (6)	
C6	0.29917 (11)	0.7210 (6)	0.6843 (3)	0.1074 (13)	
C7	0.34503 (10)	0.5785 (5)	0.6328 (3)	0.0770 (9)	
C8	0.40323 (12)	0.6072 (8)	0.6870 (3)	0.1103 (13)	
C9	0.44560 (14)	0.4824 (10)	0.6379 (4)	0.142 (2)	
C10	0.4307 (2)	0.3267 (10)	0.5382 (5)	0.150 (2)	
C11	0.3731 (2)	0.2940 (10)	0.4833 (4)	0.1460 (19)	
C12	0.32992 (13)	0.4217 (8)	0.5303 (3)	0.1111 (13)	
H1	−0.0229 (13)	0.297 (7)	0.505 (3)	0.135 (11)*	
HN1	0.1926 (9)	0.252 (5)	0.7727 (19)	0.066 (6)*	
H2A	0.09260	0.09990	0.77080	0.0640*	
H2B	0.05180	0.27210	0.83560	0.0640*	
H3	0.12080	0.63910	0.83810	0.0610*	
H4A	0.16100	0.18830	0.98910	0.1290*	
H4B	0.11890	0.41270	1.02020	0.1290*	
H4C	0.18490	0.48230	1.01930	0.1290*	
H6A	0.31780	0.82000	0.75840	0.1290*	
H6B	0.27740	0.84750	0.62410	0.1290*	
H8	0.41460	0.71210	0.75800	0.1320*	
H9	0.48550	0.50720	0.67510	0.1710*	
H10	0.45980	0.24110	0.50670	0.1790*	
H11	0.36230	0.18520	0.41350	0.1750*	
H12	0.29020	0.39990	0.49140	0.1330*	

### Atomic displacement parameters (Å^2^)

**Table:**

	*U*^11^	*U*^22^	*U*^33^	*U*^12^	*U*^13^	*U*^23^
O1	0.0694 (9)	0.0549 (8)	0.0760 (10)	−0.0139 (7)	0.0005 (7)	0.0066 (7)
O2	0.0658 (9)	0.0513 (8)	0.0775 (10)	−0.0062 (7)	0.0023 (7)	0.0186 (7)
O3	0.0803 (11)	0.0347 (7)	0.1441 (16)	0.0014 (7)	0.0394 (10)	−0.0024 (8)
O4	0.0618 (8)	0.0487 (8)	0.1039 (11)	−0.0028 (6)	0.0369 (8)	0.0035 (8)
N1	0.0533 (9)	0.0339 (8)	0.0699 (10)	0.0031 (7)	0.0207 (7)	−0.0012 (7)
C1	0.0456 (9)	0.0415 (9)	0.0642 (11)	0.0040 (8)	0.0145 (8)	0.0028 (9)
C2	0.0532 (10)	0.0466 (10)	0.0626 (12)	0.0030 (8)	0.0162 (9)	0.0091 (9)
C3	0.0533 (10)	0.0466 (10)	0.0551 (11)	0.0056 (8)	0.0161 (8)	−0.0010 (8)
C4	0.0801 (15)	0.119 (2)	0.0582 (13)	0.0027 (16)	0.0129 (11)	0.0025 (14)
C5	0.0518 (10)	0.0385 (10)	0.0696 (12)	0.0031 (8)	0.0119 (9)	0.0036 (9)
C6	0.0802 (16)	0.0665 (16)	0.194 (3)	−0.0051 (13)	0.0697 (19)	0.0239 (19)
C7	0.0555 (12)	0.0749 (16)	0.1052 (19)	−0.0028 (11)	0.0281 (12)	0.0230 (15)
C8	0.0677 (16)	0.140 (3)	0.124 (2)	−0.0100 (18)	0.0234 (16)	−0.005 (2)
C9	0.0618 (17)	0.180 (4)	0.190 (4)	0.009 (2)	0.038 (2)	0.004 (3)
C10	0.123 (3)	0.147 (4)	0.212 (5)	0.000 (3)	0.108 (3)	−0.013 (3)
C11	0.148 (3)	0.170 (4)	0.142 (3)	−0.040 (3)	0.079 (3)	−0.037 (3)
C12	0.0780 (18)	0.142 (3)	0.113 (2)	−0.017 (2)	0.0209 (17)	0.007 (2)

### Geometric parameters (Å, °)

**Table:**

O1—C1	1.301 (2)	C9—C10	1.328 (7)
O2—C1	1.231 (2)	C10—C11	1.344 (7)
O3—C5	1.208 (2)	C11—C12	1.381 (6)
O4—C5	1.350 (2)	C2—H2A	0.9700
O4—C6	1.438 (3)	C2—H2B	0.9700
O1—H1	1.18 (3)	C3—H3	0.9800
N1—C3	1.459 (2)	C4—H4A	0.9600
N1—C5	1.322 (3)	C4—H4B	0.9600
N1—HN1	0.85 (2)	C4—H4C	0.9600
C1—C2	1.483 (3)	C6—H6A	0.9700
C2—C3	1.515 (3)	C6—H6B	0.9700
C3—C4	1.515 (3)	C8—H8	0.9300
C6—C7	1.492 (4)	C9—H9	0.9300
C7—C12	1.356 (5)	C10—H10	0.9300
C7—C8	1.353 (4)	C11—H11	0.9300
C8—C9	1.372 (5)	C12—H12	0.9300
			
O1···C2^i^	3.357 (2)	H1···O1^iii^	2.74 (3)
O1···O1^ii^	3.156 (2)	H1···O2^iii^	1.48 (3)
O1···O2^iii^	2.650 (2)	H1···C1^iii^	2.38 (3)
O2···O1^iii^	2.650 (2)	H1···H1^iii^	2.29 (5)
O2···N1	3.188 (2)	HN1···O3^vii^	2.04 (2)
O2···C1^iii^	3.412 (2)	HN1···H2A	2.4300
O3···N1^iv^	2.865 (2)	H2A···HN1	2.4300
O1···H2B^i^	2.7500	H2A···H3^vii^	2.4500
O1···H1^iii^	2.74 (3)	H2B···H4B	2.3800
O2···H2B^v^	2.8300	H2B···O1^v^	2.7500
O2···H3	2.5900	H2B···O2^i^	2.8300
O2···H1^iii^	1.48 (3)	H2B···C1^i^	3.0000
O3···HN1^iv^	2.04 (2)	H3···O2	2.5900
O3···H3	2.4600	H3···O3	2.4600
O3···H6A	2.6200	H3···H2A^iv^	2.4500
O3···H6B	2.6400	H4B···H2B	2.3800
O3···H12^vi^	2.9200	H4B···C1^ix^	2.9100
O4···H12	2.7700	H4C···H6B^vi^	2.3500
N1···O2	3.188 (2)	H6A···O3	2.6200
N1···O3^vii^	2.865 (2)	H6A···H8	2.3000
C1···O2^iii^	3.412 (2)	H6B···O3	2.6400
C2···O1^v^	3.357 (2)	H6B···H4C^x^	2.3500
C1···H2B^v^	3.0000	H8···H6A	2.3000
C1···H1^iii^	2.38 (3)	H12···O4	2.7700
C1···H4B^viii^	2.9100	H12···O3^x^	2.9200
			
C5—O4—C6	115.96 (18)	C3—C2—H2B	108.00
C1—O1—H1	116.0 (16)	H2A—C2—H2B	107.00
C3—N1—C5	122.56 (16)	N1—C3—H3	108.00
C5—N1—HN1	117.3 (15)	C2—C3—H3	108.00
C3—N1—HN1	118.9 (15)	C4—C3—H3	108.00
O1—C1—O2	122.37 (18)	C3—C4—H4A	109.00
O1—C1—C2	114.36 (17)	C3—C4—H4B	109.00
O2—C1—C2	123.24 (18)	C3—C4—H4C	109.00
C1—C2—C3	115.92 (16)	H4A—C4—H4B	110.00
C2—C3—C4	110.66 (17)	H4A—C4—H4C	109.00
N1—C3—C2	110.09 (15)	H4B—C4—H4C	109.00
N1—C3—C4	111.15 (17)	O4—C6—H6A	110.00
O3—C5—N1	125.75 (19)	O4—C6—H6B	110.00
O3—C5—O4	123.51 (18)	C7—C6—H6A	110.00
O4—C5—N1	110.74 (17)	C7—C6—H6B	110.00
O4—C6—C7	107.4 (2)	H6A—C6—H6B	109.00
C6—C7—C8	120.2 (3)	C7—C8—H8	120.00
C6—C7—C12	121.4 (3)	C9—C8—H8	120.00
C8—C7—C12	118.4 (3)	C8—C9—H9	119.00
C7—C8—C9	120.4 (3)	C10—C9—H9	119.00
C8—C9—C10	121.2 (4)	C9—C10—H10	120.00
C9—C10—C11	119.4 (4)	C11—C10—H10	120.00
C10—C11—C12	120.1 (4)	C10—C11—H11	120.00
C7—C12—C11	120.5 (3)	C12—C11—H11	120.00
C1—C2—H2A	108.00	C7—C12—H12	120.00
C1—C2—H2B	108.00	C11—C12—H12	120.00
C3—C2—H2A	108.00		
			
C6—O4—C5—O3	−1.5 (3)	O4—C6—C7—C8	−122.5 (3)
C6—O4—C5—N1	178.82 (19)	O4—C6—C7—C12	58.6 (4)
C5—O4—C6—C7	179.6 (2)	C6—C7—C8—C9	−178.2 (3)
C5—N1—C3—C2	139.00 (18)	C12—C7—C8—C9	0.7 (5)
C5—N1—C3—C4	−98.0 (2)	C6—C7—C12—C11	179.3 (3)
C3—N1—C5—O3	−4.8 (3)	C8—C7—C12—C11	0.3 (5)
C3—N1—C5—O4	174.88 (16)	C7—C8—C9—C10	−1.4 (7)
O1—C1—C2—C3	175.10 (16)	C8—C9—C10—C11	1.0 (8)
O2—C1—C2—C3	−6.8 (3)	C9—C10—C11—C12	0.1 (7)
C1—C2—C3—N1	−73.0 (2)	C10—C11—C12—C7	−0.8 (7)
C1—C2—C3—C4	163.72 (18)		

Symmetry codes: (i) −*x*, *y*−1/2, −*z*+3/2; (ii) −*x*, −*y*, −*z*+1; (iii) −*x*, −*y*+1, −*z*+1; (iv) *x*, *y*+1, *z*; (v) −*x*, *y*+1/2, −*z*+3/2; (vi) *x*, −*y*+3/2, *z*+1/2; (vii) *x*, *y*−1, *z*; (viii) *x*, −*y*+1/2, *z*−1/2; (ix) *x*, −*y*+1/2, *z*+1/2; (x) *x*, −*y*+3/2, *z*−1/2.

### Hydrogen-bond geometry (Å, °)

**Table:**

*D*—H···*A*	*D*—H	H···*A*	*D*···*A*	*D*—H···*A*
O1—H1···O2^iii^	1.18 (3)	1.48 (3)	2.650 (2)	177 (2)
N1—HN1···O3^vii^	0.85 (2)	2.04 (2)	2.865 (2)	165 (2)
C3—H3···O3	0.98	2.46	2.825 (3)	101.

Symmetry codes: (iii) −*x*, −*y*+1, −*z*+1; (vii) *x*, *y*−1, *z*.
